# Frémy’s Salt as a Low-Persistence Hyperpolarization Agent: Efficient Dynamic Nuclear Polarization Plus Rapid Radical Scavenging

**DOI:** 10.1021/jacs.2c07960

**Published:** 2022-11-02

**Authors:** Mattia Negroni, Ertan Turhan, Thomas Kress, Morgan Ceillier, Sami Jannin, Dennis Kurzbach

**Affiliations:** † Faculty of Chemistry, Institute of Biological Chemistry, University Vienna, Währinger Straße 38, 1090 Vienna, Austria; ‡ Yusuf Hamied Department of Chemistry, University of Cambridge, Lensfield Road, Cambridge, CB2 1EW, U.K.; § Centre de Résonance Magnétique Nucléaire à Très Hauts Champs (UMR 5082) Université de Lyon/CNRS/Université Claude Bernard Lyon 1/ENS de Lyon, 5 Rue de la Doua, 69100 Villeurbanne, France

## Abstract

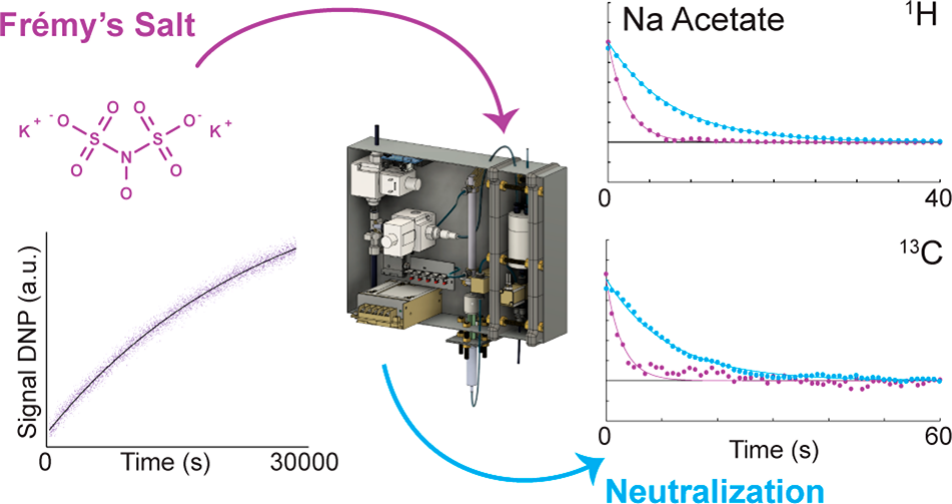

Nuclear magnetic resonance (NMR) spectroscopy is a key technique for molecular structure determination in solution. However, due to its low sensitivity, many efforts have been made to improve signal strengths and reduce the required substrate amounts. In this regard, dissolution dynamic nuclear polarization (DDNP) is a versatile approach as signal enhancements of over 10 000-fold are achievable. Samples are signal-enhanced *ex situ* by transferring electronic polarization from radicals to nuclear spins before dissolving and shuttling the boosted sample to an NMR spectrometer for detection. However, the applicability of DDNP suffers from one major drawback, namely, paramagnetic relaxation enhancements (PREs) that critically reduce relaxation times due to the codissolved radicals. PREs are the primary source of polarization losses canceling the signal improvements obtained by DNP. We solve this problem by using potassium nitrosodisulfonate (Frémy’s salt) as polarization agent (PA), which provides high nuclear spin polarization and allows for rapid scavenging under mild reducing conditions. We demonstrate the potential of Frémy’s salt, (i) showing that both ^1^H and ^13^C polarization of ∼30% can be achieved and (ii) describing a hybrid sample shuttling system (HySSS) that can be used with any DDNP/NMR combination to remove the PA before NMR detection. This gadget mixes the hyperpolarized solution with a radical scavenger and injects it into an NMR tube, providing, within a few seconds, quantitatively radical-free, highly polarized solutions. The cost efficiency and broad availability of Frémy’s salt might facilitate the use of DDNP in many fields of research.

## Introduction

Dynamic nuclear polarization (DNP) is a hyperpolarization technique aiming to boost nuclear magnetic resonance (NMR) signals and overcome their intrinsically weak intensities. DNP is based on transferring the high magnetization of unpaired electrons to nuclei in their vicinities^1^ by microwave irradiation at low temperatures.^2^ DNP applications for high-field solution-state NMR is possible via the dissolution DNP (DDNP)^3^ approach. The sample is hyperpolarized at low temperatures and rapidly dissolved before being injected into an NMR magnet.^3^ Applicable to a wide array of target molecules, from small metabolites^4−8^ to nucleic acids,^9^ proteins,^10−13^ and inorganic ions,^14^ DDNP is an asset in many research programs, from preclinical imaging,^15,16^ to cancer screening^17,18^ and analytical applications in chemistry^19−21^ and physics.^22,23^

However, despite its versatility and regularly achieved signal enhancements of >10 000-fold,^3^ this method suffers from one main drawback: the radical providing the unpaired electron needed for DNP at low temperatures becomes an unwanted source of rapid nuclear spin relaxation at ambient temperatures in solution.^24−26^

To overcome this problem and the related paramagnetic relaxation enhancements (PREs) tremendous efforts have recently been made to scavenge the radicals after dissolution, for example, through reduction,^27^ filtration,^28,29^ solvent extraction,^30^ phase separation, electrochemical removal,^31^ or annealing of photoinduced radicals.^32^ It should be noted that, in particular, tailored polarization agents (PAs) immobilized on thermoresponsive polymers,^33^ hydrogels,^34,35^ or solid matrices^36,37^ have received ample attention, as these can be in-line filtered upon dissolution. This strategy has the advantage that no scavenging reagent is needed for quantitative radical removal. However, the availability of the special PAs is limited, and custom filters are required. Hence, PA reduction arguably remains the most apparent and ready-to-implement solution, not requiring specifically synthesized or expensive materials. However, when working with stable radicals, the neutralization kinetics are often much slower than the nuclear relaxation,^27^ imposing the use of harsh reducing conditions or large excesses of scavenger reagents. However, this solution is rarely applicable since the target molecule is likely attacked, too.

Herein, we propose a straightforward solution to the problem of paramagnetic contamination in DDNP: the well-known radical Frémy’s salt (FS). Using FS as a PA, we demonstrate that not only can ^1^H and ^13^C polarization of ∼30% be achieved at 1.4 K in generic solutions but also that quantitative radical scavenging is possible within a few seconds with only 1 equiv of a mild reducing agent such as sodium ascorbate. Hence, with this broadly available PA, the performance (in terms of maximal low-temperature hyperpolarization) of current cutting-edge PAs can be matched while providing the additional possibility of simple radical removal.

Due to its widespread use in synthetic chemistry, FS is a cost-efficient and readily available PA that renders DNP experiments effective and economical. At the same time, it provides the potential to simultaneously polarize ^1^H and ^13^C nuclei, rendering FS applicable to a comprehensive set of target molecules. Furthermore, with FS, radical-free DDNP-boosted NMR becomes possible for reduction-sensitive compounds, such as proteins containing Cys residues or oxidation agents, due to very mild scavenging conditions while not requiring any specialized designer PAs or a customized filter.

## Results and Discussion

Potassium nitrosodisulfonate (K_2_[NO(SO_3_)_2_]), i.e., FS, is a well-established radical in electron paramagnetic resonance (EPR) applications and oxidative reactions.^38^ It was also successfully used as PA for solution-state Overhauser DNP at high magnetic fields.^39−41^ However, the use of FS in low-temperature DNP has not been reported yet. We benchmarked its DNP capabilities at 1.4 K using a solution of 1.5 M sodium pyruvate-1-^13^C supplemented with 40 mM of Frémy’s salt in glycerol-*d*_8_:D_2_O:H_2_O 5:4:1 (v/v). The DNP setup operating at a magnetic field of *B*_0,DNP_ = 6.7 T is described in ref (42).

We achieved high DNP ^13^C enhancement upon continuous microwave irradiation at 188.112 GHz. The extrapolated steady-state polarization at *t* → ∞ reached *P*(^13^C) = 31 ± 7% at an FS concentration of 40 mM. (The values extrapolated for *t* → ∞ are buildup time independent. Hence, they render the polarization comparison more general as values taken at one specific time point.) Next, we compared these results to the “gold standard” OX063, a triaryl methyl (trityl) radical. At a typical concentration of 15 mM, we found a steady-state polarization of *P*(^13^C) = 17 ± 8% in our home-built DNP system, agreeing qualitatively with a previous study by Lumata et al.^43^ when taking the differences in experimental magnetic fields (3.3 vs 6.7 T) into account. Higher concentrations of 40 mM led to a faster and better polarization of *P*(^13^C) = 21 ± 5%. Note that for neat pyruvic-1-^13^C acid, carbon polarization up to *P*(^13^C) > 70% can be achieved with OX063.^44−48^ With another nitroxide, namely, 40 mM TEMPOL (as often used in multicontact cross-polarization DNP),^49^ the polarization plateaued at only 1 ± 0.3% (see the Supporting Information Figure S1).

Summarizing the above, not only is FS a better polarizing agent for carbon-13 nuclei compared to TEMPOL, but the polarization can compare with that of the “gold standard” OX063 for generic solutions of a target molecule, albeit with slower buildup kinetics (see the Supporting Information for the kinetics data).

Besides, FS provides the additional benefit of enhancing proton polarizations to a substantial degree. We achieved a proton polarization of 29 ± 5% by direct irradiation at 187.912 GHz. With TEMPOL we obtained 48 ± 5% at 1.4 K (without microwave modulation).^50−52^ Hence, FS can be used for direct ^13^C hyperpolarization as well as multicontact ^1^H–^13^C cross-polarization-based DNP.^49^ OX063 was not found efficient for proton DNP in our hands. Table 1 lists all reported polarizations.

**Table 1:** Solid-State ^1^H and ^13^C Polarizations Obtained with Different PA on 1.5 M Sodium Pyruvate-1-^13^C Samples at 1.4 K and 6.7 T at *t* → ∞^a^

PA	*P*(^1^H)	*P*(^13^C)
FS (40 mM)	29 ± 5%	31 ± 7%
OX063 (15 mM)	1 ± 0.1%	17 ± 8%
OX063 (40 mM)	1 ± 0.1%	21 ± 5%
TEMPOL (40 mM)	48 ± 5%	1 ± 0.3%

a The reported errors are mainly due to uncertainties in the evaluation of thermal equilibrium signal intensities at 1.4 K (see the Supporting Information for details and a systematic error analysis).

It should be noted that other absolute steady-state polarizations can be achieved with other DNP systems featuring, for example, lower base temperatures or microwave delivery systems.^53^ However, relative comparisons between PAs under similar conditions are not influenced by such differences.

Interestingly, the DNP-frequency profile (Figure S4) of FS is markedly different from those of other nitroxides like TEMPOL.^54,55^ The positive lobe is significantly more intense than the negative one. This might explain the good DNP performance of FS.

**Figure 1:**
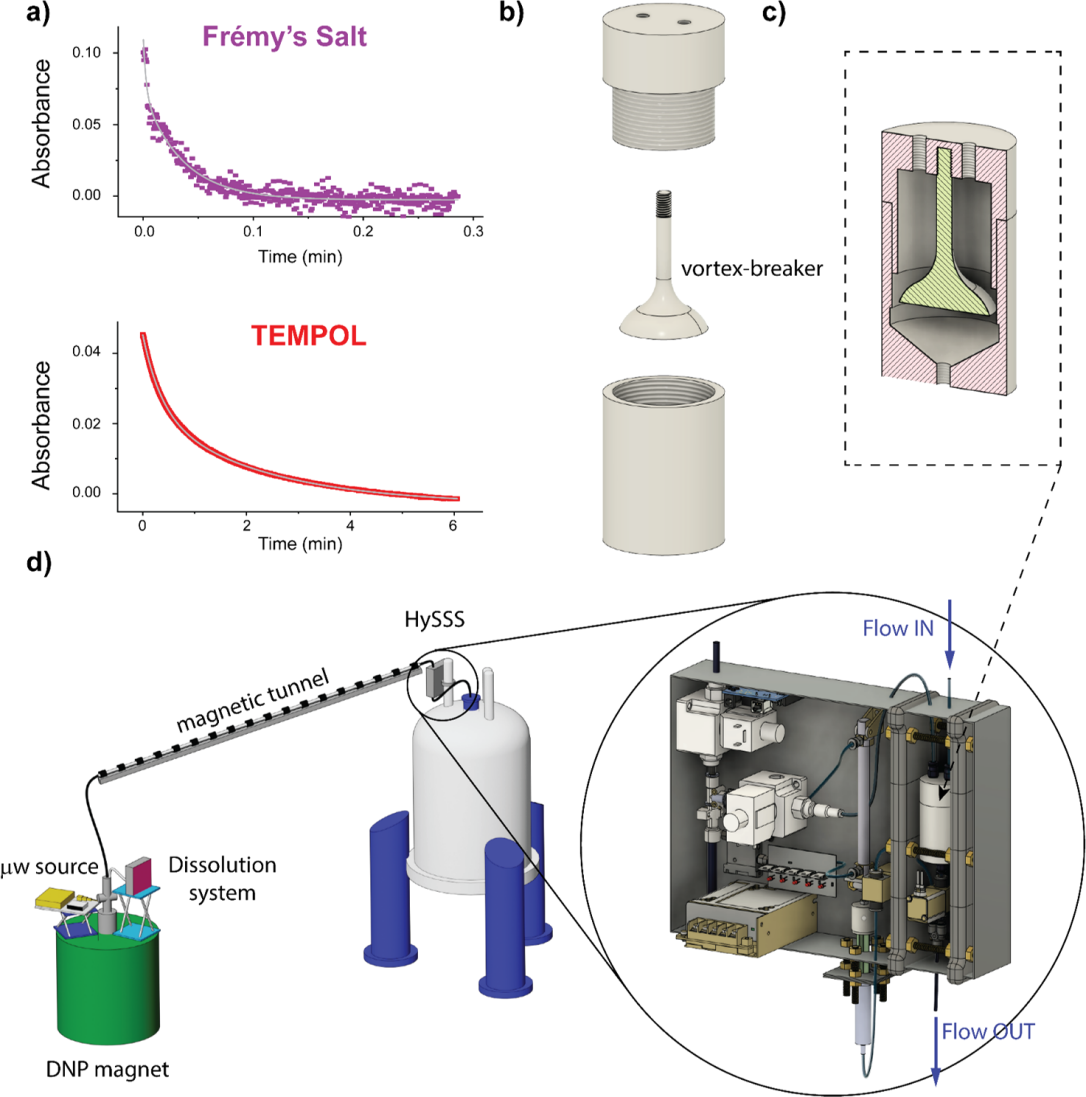
(a) UV time traces for neutralization reactions of Frémy’s salt and TEMPOL using sodium ascorbate; absorbance monitored at 543 and 428 nm, respectively (Figure S5). Biexponential fits are shown in gray. Frémy’s salt is quenched ca. 100 times faster than TEMPOL. (b) Disassembled view of the individual collector/mixing chamber components. The vortex breaker is essential to avoid air inclusions. (c) Section of the assembled mixing chamber in which the radical is neutralized before injection in the NMR tube. (d) Representation of the DDNP system with a detailed drawing of the HySSS (details in the Supporting Information). The mixing chamber is visible between two gray magnetic plates on the right site of the case.

After low-temperature DNP buildup, we tested the neutralization kinetics of FS upon dissolution. In powder form, the radical is stable, but dissolved in water it becomes particularly reactive. We monitored the neutralization by time-resolved UV/vis spectroscopy using a 5 mM FS solution in water (corresponding to the conditions in a DDNP experiment after dissolution at pH 7) with 1 equiv of sodium ascorbate. For FS, we found a biexponential decay with time constants of 0.15 ± 0.03 s and 2.14 ± 0.08 s. 95% of the radicals were reduced within 5 s. For comparison, the same experiment performed with TEMPOL resulted in time constants of 21.48 ± 0.05 s and 131.5 ± 0.2 s (Figure 1a). Hence, on the time scale of a DDNP experiment, where detection must start a few seconds after dissolution, FS can be quantitatively scavenged due to its low persistence under mild reducing conditions. (The very high reactivity of FS must also be considered when preparing the DNP sample. FS degrades within minutes when the DNP samples are left at room temperature.)

However, combining the dissolution step of a DDNP experiment with mixing FS and a reducing agent is challenging, particularly when precise control over the experiment is desired. Indeed, the dissolution event is often chaotic due to the speed of the process (typically only a few seconds from dissolution to injection into the NMR spectrometer). Previously, frozen beads of a DNP sample and of a neutralizing solution were cofrozen; mixing was then achieved by the simultaneous dissolution of both beads.^27^ However, sample preparation becomes complex, and control over mixing both components during the melting process is complicated to establish. To improve control and simplify the scavenging process, we designed a 40 mm diameter sample collector, which degasses the dissolved hyperpolarized solution and concertedly mixes it with the reducing agent (Figure 1b,c). The collector is embedded in a hybrid pneumatic/hydraulic sample shuttling system (HySSS; Figure 1d) that transfers radical-free solutions into the NMR tube for detection. Mixing and sample shuttling take place in three steps: 1. The incoming hyperpolarized sample (at ca. 7 bar chase gas pressure) accumulates in the collector, where a neutralizing solution is waiting. 2. After mixing both solutions, the sample is degassed by a vacuum line for 750 ms before being pushed out of the collector. A “vortex breaker” (Figure 1b) guarantees that the chase gas is not reintroducing any bubbles after the degassing step. The collector is positioned between two magnetic plates to ensure a magnetic field of >10 mT during mixing and degassing (Figure 1d). 3. After leaving the collector/mixing chamber, the sample is hydraulically (to avoid gas inclusions) pushed through a capillary into the NMR tube, waiting *in situ* in the NMR spectrometer, ready for detection. The volume of the hydraulic driving liquid thereby precisely controls the injection volume. The entire process takes <2.5 s. The HySSS is described in detail in the Supporting Information.

The HySSS solves three problems: (i) Experimental control: Mixing of the hyperpolarized sample with the D_2_O used to dissolve it is typically not homogeneous (a strong concentration gradient is generally observed along the liquid bolus traveling toward the NMR spectrometer). Hence, FS reduction through adding ascorbate to the dissolution solvent would lead to uncontrolled experimental conditions. Within the HySSS homogeneous mixing with the entire hyperpolarized solution is guaranteed. (ii) Low scavenger concentrations: The homogeneous mixing minimizes the necessary quantities of the reducing agent. Other approaches would require much higher concentrations to guarantee quantitative FS reduction. (iii) Mild conditions: The decomposition temperature of ascorbate is typically below that of the used dissolution solvent (190 °C vs 220 °C). Hence, adding ascorbate to the dissolution solvent directly leads to the presence of contaminant side products. Within the HySSS, the scavenger solution waits at room temperature.

Note that other reported injectors for DDNP experiments often depend on multigate valves and liquid collection loops.^57−59^ Hence, most previously reported “mixing” experiments were conducted by mixing the arriving hyperpolarized solution with the target solution directly in the NMR tube used for detection downstream to volume control and optional degassing.^30,60^ The HySSS is fundamentally different, using a collection chamber with a dedicated vortex breaker to degas the sample, control the injection, and mix two solutions in a single step.

**Figure 2:**
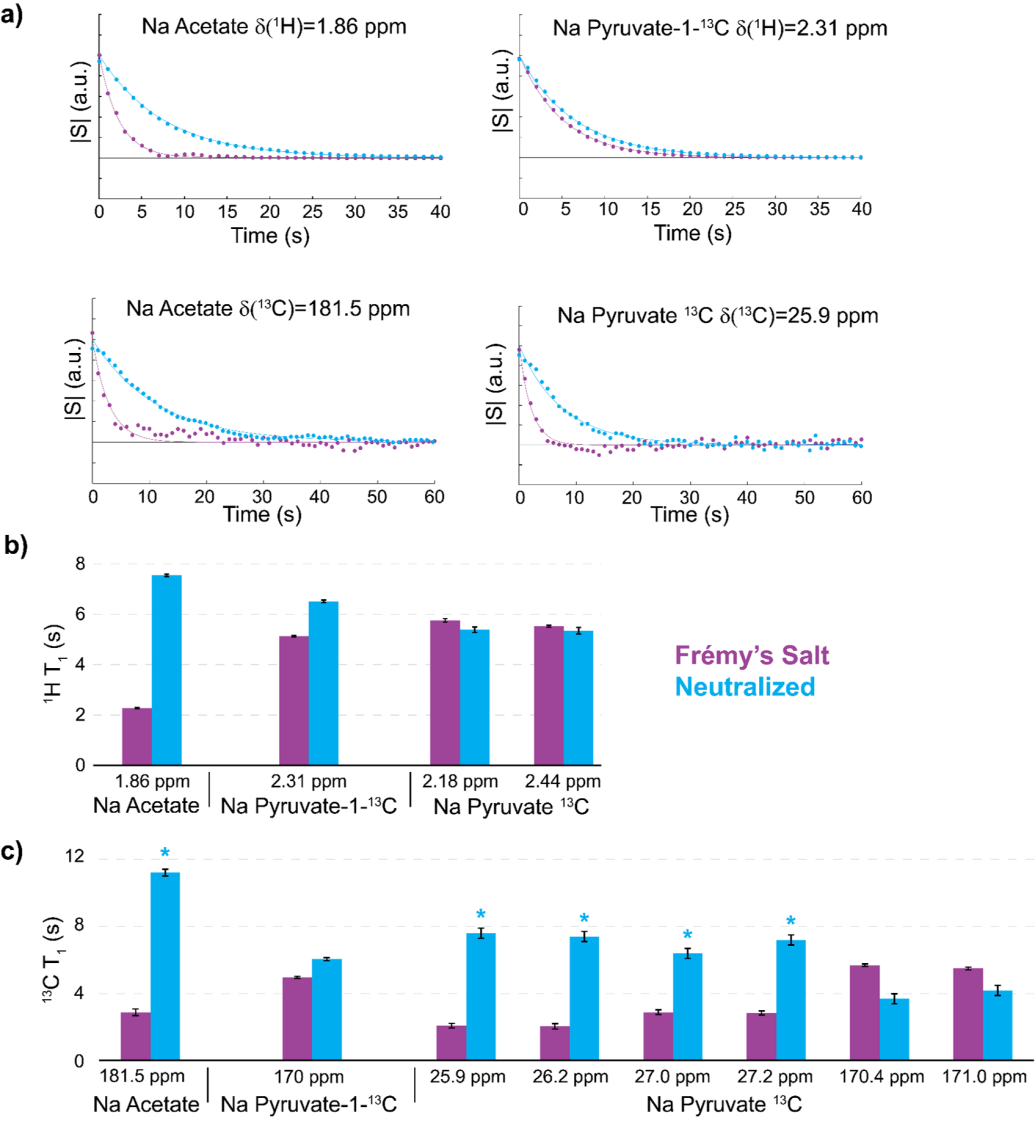
Longitudinal relaxation times of the various observable spin species after injection in the detection spectrometer. Purple curves refer to experiments without any neutralization (*B*_0_ = 11.7 T), while blue ones to experiments employing radical scavenging (for the full spectra, see Figure S6). (a) Example of polarization decays with (blue) and without (purple) neutralization of FS (for the other signals, see Figure S7). (b) Proton longitudinal relaxation times with (blue) and without (purple) neutralization of FS. (c) The same as in panel b, but for carbon-13 longitudinal relaxation times (asterisks indicate signals that might be affected by cross-relaxation effects).^56^

Using the HySSS, DDNP experiments by FS were performed with three different molecules: sodium acetate-1-^13^C, sodium pyruvate-1-^13^C, and uniformly ^13^C-labeled sodium pyruvate. All the samples were prepared at a concentration of 1.5 M in glycerol-*d*_8_:D_2_O:H_2_O at a volumetric ratio of 5:4:1. Upon injection into the NMR spectrometer, the hyperpolarized signals were detected in parallel for ^1^H and ^13^C every second at a magnetic field of 11.7 T. Representative signal decay curves after injection are shown in Figure 2a.

In almost all cases, the relaxation times could efficiently be prolonged by neutralizing the radical (blue curves) compared to similar experiments without radical scavenging (purple curves). The decays were fitted to exponential functions, and the resulting *T*_1_ times are shown in Figure 2b for ^1^H and Figure 2c for ^13^C nuclei. The most pronounced effects were observed for the protons of sodium acetate, from *T*_1_ = 2.3 s to 7.5 s. A prolongation of 1.5 s was observed for pyruvate-1-^13^C. The proton spins of fully enriched sodium pyruvate were interestingly unaffected. Concerning ^13^C, the neutralization strongly influences protonated moieties, with *T*_1_ being 3- to 4-fold longer. Only signals of quaternary carbons remained unaffected. The line-broadening of the enhanced signals was dominated by injection inhomogeneities (e.g., microbubbles),^57^ such that paramagnetic relaxation enhancement was not the major source of line broadening in our experiments. As a result, radical scavenging did not strongly influence the line shape.

The solution-state enhancements were on the order of 10 000 for ^13^C and 100 for ^1^H nuclei. A significant fraction of the polarization was lost during sample dissolution and transfer due to fast relaxation during rapid low-field passage.^61^ It should be noted, though, that solution-state enhancements depend critically on the layout of the laboratory where the DDNP system is situated and the effective magnetic fields during sample transfer.^62^

The 3- to 4-fold prolonged relaxation times enable a similarly extended detection time window. This feature might be useful, for example, in DDNP experiments aiming to detect metabolic conversion of hyperpolarized substrates. Indeed, many metabolites appear only minutes after the uptake of a hyperpolarized starting material by either an enzymatic cocktail or a cell or even upon injection into living organisms.^63−67^ FS might be a versatile asset to improve the sensitivity for delayed metabolic processes.

At the same time, comparable advantages can be expected for monitoring chemical reactions,^68^ materials formation,^69^ or detecting hyperpolarized 2D and 3D spectra^70,71^ or chemical exchange events.^72^ The sets of reactions, reaction conditions, interactions, and conversions are significantly widened with a 3- to 4-fold longer detection time window.

On the contrary, it should be noted that before aiming to use FS for *in vivo* experiments in animals or humans, the toxicity of the reduction products needs to be determined first and a rapid quality control procedure of the hyperpolarized solution needs to be established (for OX063 this takes only 30 s).

Concluding, Frémy’s salt provides favorable properties for dissolution DNP experiments. Its ability to efficiently polarize carbons and protons is useful for double detection experiments and renders it a cost-efficient alternative for ^13^C DNP. Furthermore, FS is very reactive, allowing for swift neutralization and radical-free DDNP-boosted NMR samples with long hyperpolarization lifetimes. Using the HySSS, a functional device for controlled mixing and sample shuttling, quantitative neutralization and reproducibility of the FS-DDNP experiments are guaranteed.

## Supporting Information Available

Buildup curves, decay curves, experimental details, control experiments, explanation of the HySSS (PDF)
Data availability All raw data are available under DOI 10.5281/zenodo.7261376.
