# Supplementary material for: Frémy’s Salt as a Low-Persistence Hyperpolarization Agent: Efficient Dynamic Nuclear Polarization Plus Rapid Radical Scavenging
Source: J Am Chem Soc. 2022 Nov 2;144(45):20680–6. doi: 10.1021/jacs.2c07960 (PMC9673139; doi:10.1021/jacs.2c07960)
Supplement: Supplementary file 1 — ja2c07960_si_001.pdf [file ja2c07960_si_001.pdf]

Frémy's salt as a low-persistence hyperpolarization agent - efficient dynamic nuclear polarization plus rapid radical scavenging

Mattia Negroni†, Ertan Turhan†, Thomas Kress, Morgan Ceillier, Sami Jannin, Dennis Kurzbach*

†these authors contribute equally

-Supporting Information-

Methods

Adsorption Measures

The neutralization reactions were monitored by detecting the main adsorption peak (FS: 543 nm, TEMPLO: 428 nm, Figure S1) of a 5 mM PA solution in water over time using a Cary 5000 UV-Vis-NIR spectrometer with stirring. The neutralization reaction was initiated by adding 1 equivalent of sodium ascorbate pre-dissolved in water and stirring for 20 s. Note that the data in Fig. 1a is noisier for FS than for TEMPOL, since the data had to be recorded under stirring due to the fast kinetics of the neutralization reaction. For TEMPOL, the stirrer could be switched before starting the detection.

Dissolution DNP

The DDNP experiments were performed on three substrates: sodium acetate-1-^{13}C, sodium pyruvate-1-^{13}C, and uniformly 13C-labelled sodium pyruvate. All the samples were prepared at a concentration of 1.5 M in glycerol-d_8:D_2O:H_2O at a volumetric ratio of 5:4:1 and a total volume of 100 µL. The radical concentration was 15, 40 or 80 mM and dissolutions were performed after 2 h of signal build-up at a magnetic field of 6.7 T and a temperature of 1.4 K as described in reference [1] using 5 mL of pressurized (to 15 bar) D_2O as dissolution solvent. The instability of FS reduces the storage life of samples. Hence, a fresh sample was prepared before every insertion in the polarizer; note that samples became completely transparent after 10 minutes at room temperature. Fast FS neutralization was achieved through quantitative mixing of 200 µL of a sodium ascorbate solution in D_2O (Sodium ascorbate concentrations of 20 to 120 mM were tested, yielding molar ratios of 1:1 to 1:6 between FS and ascorbate ratio. However, no significant differences were observed in the DDNP experiments.) waiting in the HySSS with the ~5 mL hyperpolarized solution arriving from the DNP system. The ^{1}H and ^{13}C signals were detected simultaneously once per second on a Bruker NEO 500 MHz spectrometer equipped with a BBFO Prodigy cryogenic probe head, using 1 and 10° flip angles, respectively, as described in reference [2].

Low-temperature DNP to determine solid-state polarizations

The setup was the same as for DDNP, except for using a Teflon sample cup to reduce the background signal. The build-up and decay curves were fitted using exponential functions. The ratio between the extrapolated values at infinite time yielded the enhancements:

$$\varepsilon = \frac{\left[S_{max}\left(1-e^{-R_{build-up}\cdot n\cdot dt}\right)\right]\cos^n\theta}{\left[(S_{max}-S_{TE})e^{-R_1\cdot n\cdot dt}+S_{TE}\right]\cos^n\theta} \qquad \text{(eq. S1)}$$

$$P_n = P_{TE}\varepsilon \qquad \text{(eq. S2)}$$

$$P_{TE} = \tanh\left(\frac{\omega\hbar}{2k_BT}\right) \qquad \text{(eq. S3)}$$

Where ε is the signal enhancement, θ the detection flip angle, n the number of pulses, i.e., detected points, and dt the time between two pulses. S_{max} and S_{TE} are the signal intensities in the hyperpolarized and thermal equilibrium state, respectively. $\mathrm{R_{build-up}}$ and $\mathrm{R_1}$ are the respective build-up and decay rates. P_n is the nuclear polarization and P_{TE} is the

nuclear polarization in thermal equilibrium. ω is the nuclear Larmor frequency and T the experimental temperature. The numerator in equation S1 start from zero instead of S_{TE} to take into account the presaturation sequence.

This was then multiplied by the thermal polarization of ^{1}H and ^{13}C, respectively at a magnetic field of 6.7 T and a temperature of 1.4 K (0.49 % and 0.12 %, respectively) to obtain the theoretical maximum DNP polarization.

Note that a conventional measurement of the thermal equilibrium signal would be very unreliable as it would take several weeks with FS at 1.4 K. Hence, we used the extrapolation to determine the polarization. The reported errors stem from the 95% confidence interval of these fits . Repeating the experiments several times showed identical build-up and decay curves (*vide infra*).

EPR

EPR spectra were acquired using a Bruker Magnettech ESR5000 equipped with a finger dewar filled with liquid nitrogen. The samples were the same as for DDNP. The microwave frequency was 9.4 GHz.

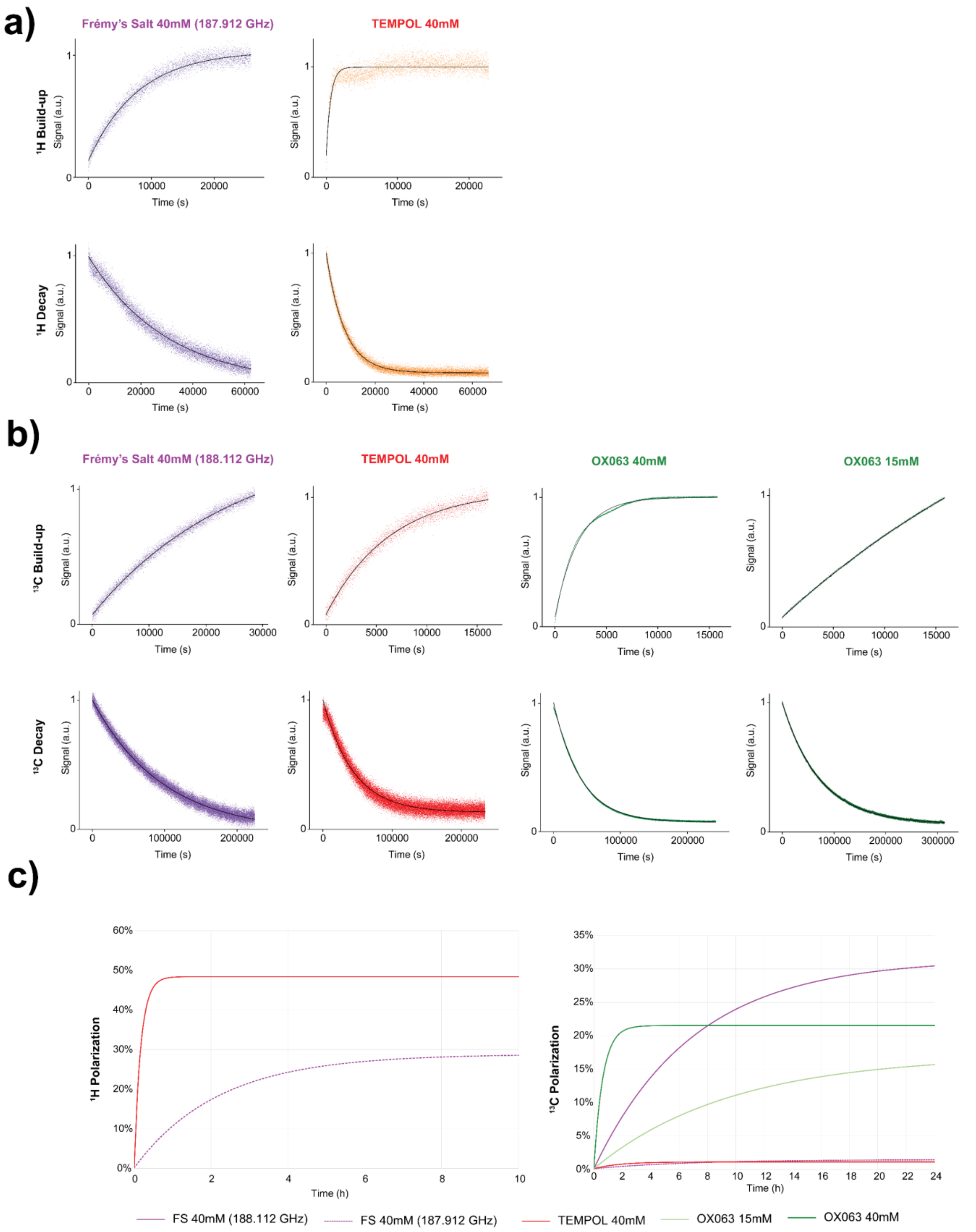

Figure S1: Results of the polarization experiments for a solution of 1.5 M pyruvate-1-^{13}C in glycerol-d_8:D_2O:H_2O 5:4:1 with different polarizing agents at 1.4 K. **a)** Proton and **b)**Carbon-13 polarization DNP build-ups and decays. Exponential fittings are shown as black lines. The pulse length was doubled for the pulse length on the OX063 experiments to speed up the decay (hence the S/N is better compared to the FS). c**)** Fitting results for the DNP build-ups normalized on the thermal signal. Note that for the build-ups, measured values lie within 2 % of the extrapolated signal intensities.

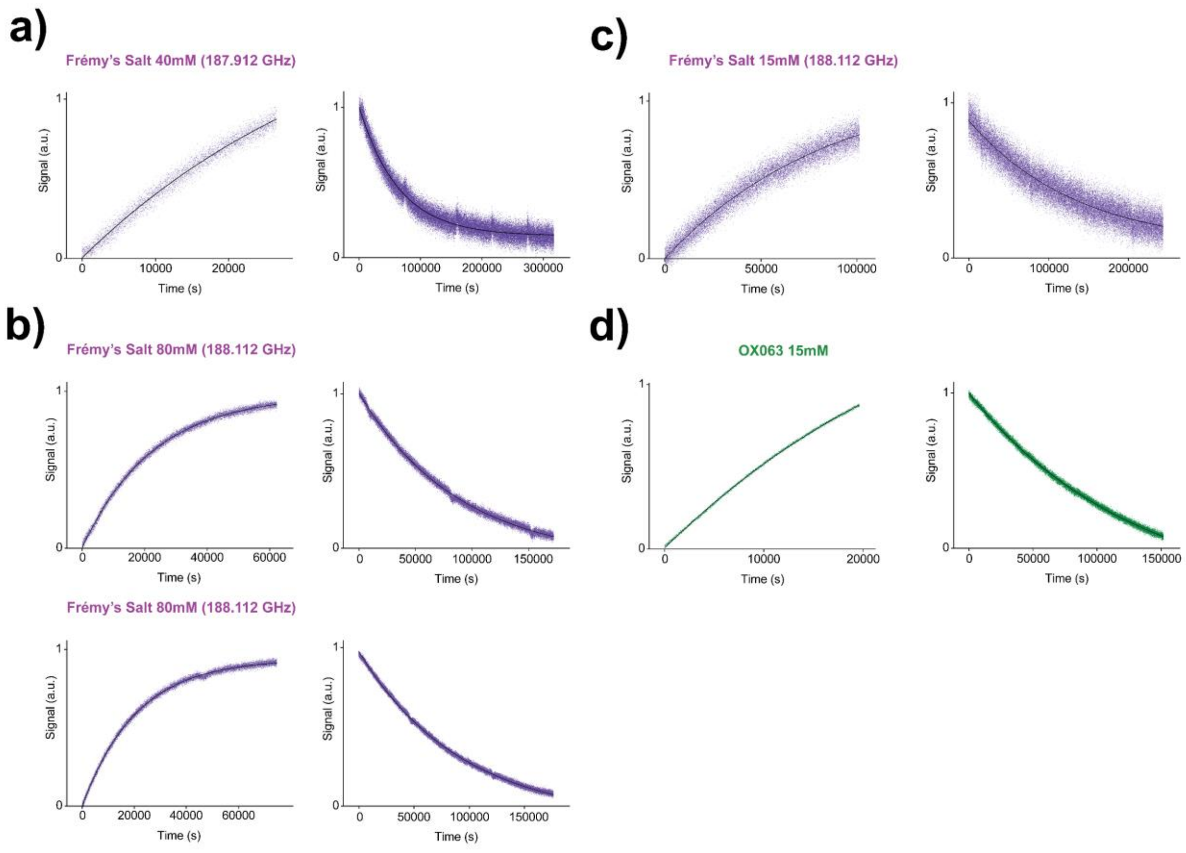

Figure S2: Control experiments and error evaluation for carbon-13 hyperpolarization for a solution of 1.5 M pyruvate-1-^{13}C in glycerol-d_8:D_2O:H_2O 5:4:1 at a temperature of 1.4 K and a magnetic field of 6.7 T. **a)** Control experiment showing that Fremy's Salt leads to carbon-13 polarization upon irradiation at frequencies different from 188.112 GHz leads to DNP, too, albeit with lesser efficiency. **b)** Fremy's Salt carbon-13 polarization with very long acquisition times and at high, 80 mM, FS concentrations to approach the steady-state. The polarization obtained in the long run was similar to the one obtained from shorter acquisition periods (35%, cf. Table S1). Repetition runs are shown, too. Repetition of the experiments led to identical results within the experimental error. **c)** Carbon-13 polarization at 15 mM FS. As expected, the obtained polarization was consistently lower than at 40 or 80 mM. **d)** 15 mM OX063 polarization determination with the same pulse length as used for Fremy's Salt and TEMPOL, the obtained polarization was similar to the one obtained at a doubled pulse length (17%) shown in Fig. S1.

Table S1: ^{13}C Polarizations obtained from different experiments at $t \rightarrow \infty$ (cf. eq. S1). The errors are due to fitting uncertainties of the thermal equilibrium signal intensity (95% confidence interval, see Fig. S1 and S2). Note that the ^{13}C polarization increases with increasing PA concentration.

	^{13}C Polarization
FS (15 mM)	1±0.4
FS (40 mM)	31±7
FS (80 mM)	35±11
TEMPOL 40 mM	1±0.3
OX063 40 mM	21±5
OX063 15 mM	17±8

Table S2: Characteristic times for proton and carbon-13 polarization DNP build-ups and decays obtained from least-square exponential fitting.

	^{1}H Build-Up (s)	^{1}H Decay (s)	^{13}C Build-Up (s)	^{13}C Decay (s)
FS 40 mM (188.112 MHz)			25200	97600
FS 40 mM (187.912 MHz)	7860	30400	38700	64700
TEMPOL 40 mM	620	7445	6330	43240
OX063 40 mM			34400	73030

OX063 15 mM			2260	40320

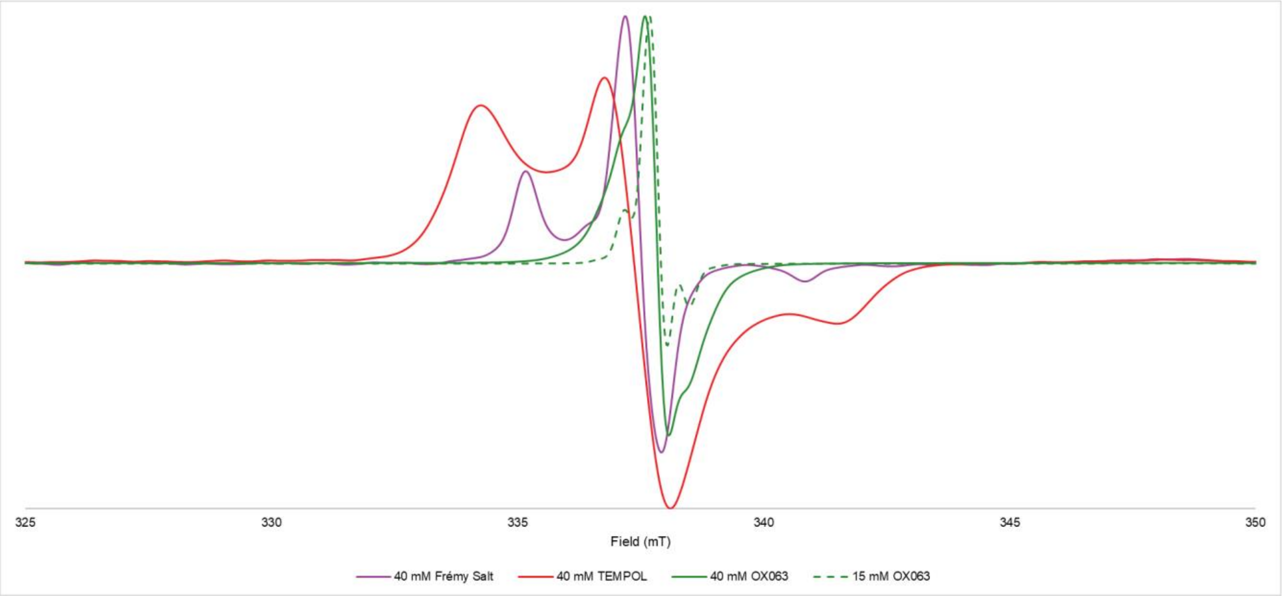

Figure S3: Continuous-wave (CW) EPR spectra acquired at 77 K for a solution of 1.5 M pyruvate-1-^{13}C in glycerol-d$_8$:D_2O:H_2O 5:4:1 with different polarizing agents.

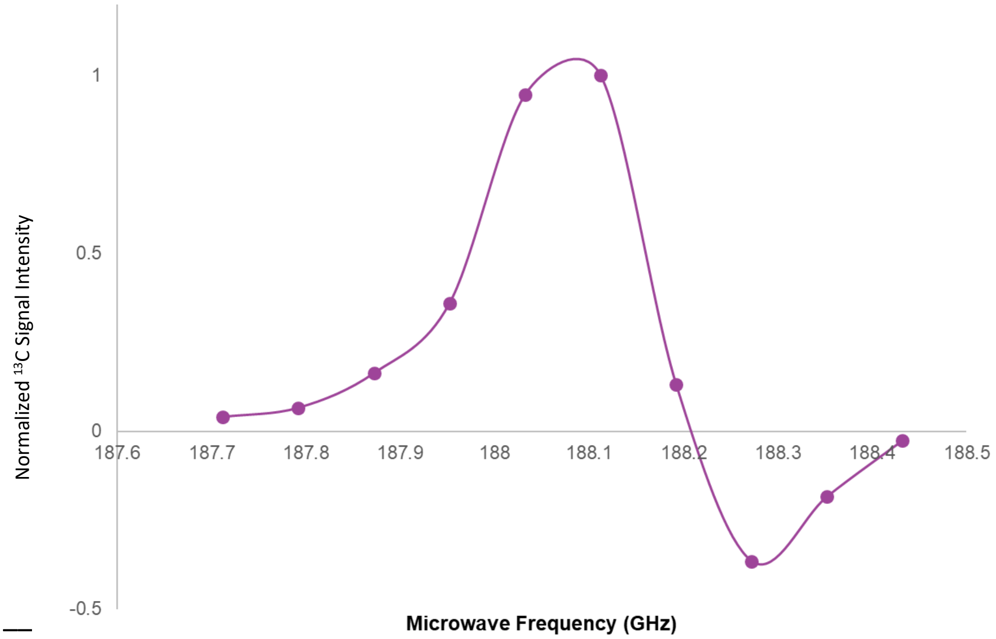

Figure S4: DNP ^{13}C frequency profile acquired at 1.4 K for a solution of 1.5 M pyruvate-1-^{13}C in glycerol-d$_8$:D_2O:H_2O 5:4:1 obtained at a FS concentration of 40 mM (each dot corresponds to the extrapolated signal strength at $t \longrightarrow \infty$). Note that the positive lob is much stronger than the negative one.

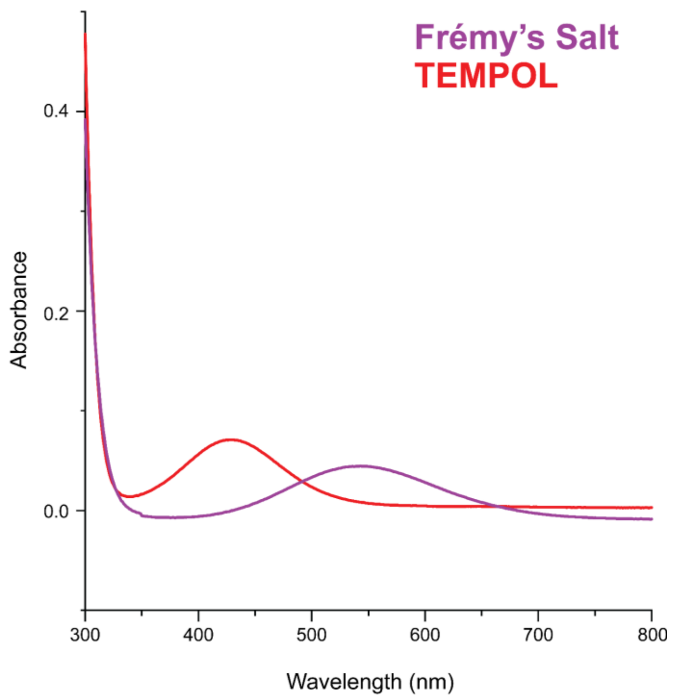

Figure S5: Adsorption spectra of 5 mM radical solutions in water.

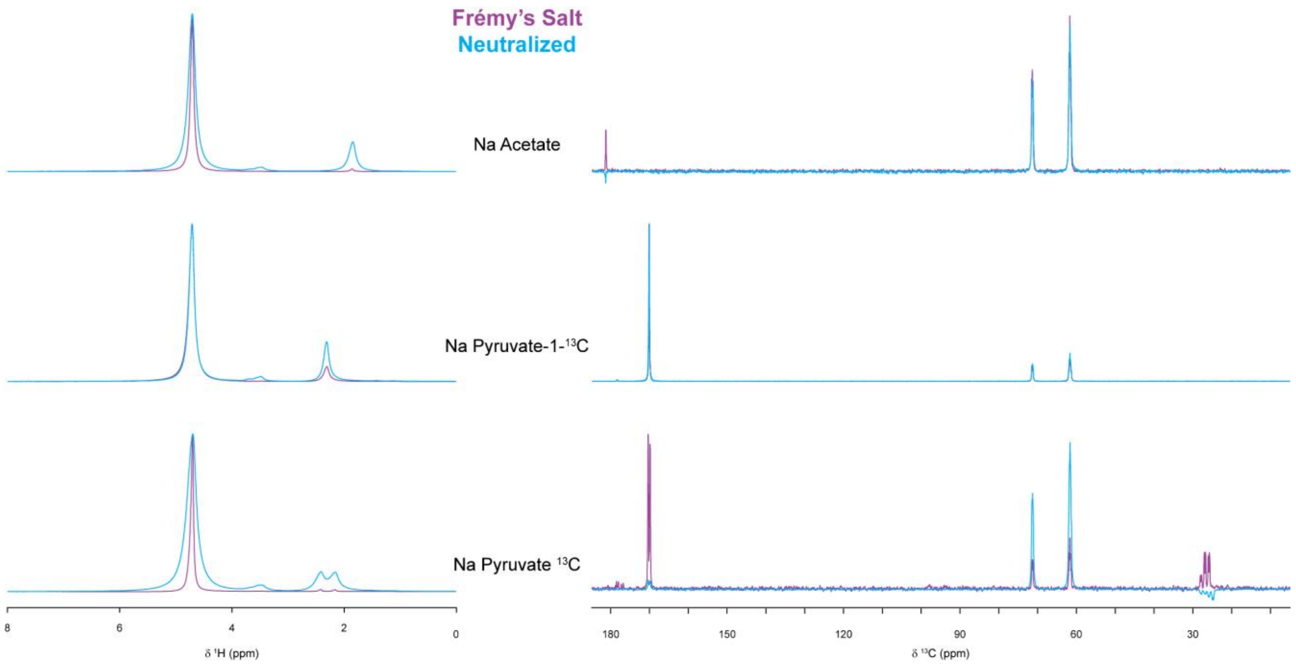

Figure S6: DDNP-boosted NMR spectra of the three substrates. Note that cross-relaxation effects can arise upon sample transfer when both ^{1}H and ^{13}C nuclei are simultaneously hyperpolarized, as we have shown in our earlier work.[19] As a result, ^{13}C signals can either be inverted or attenuated.

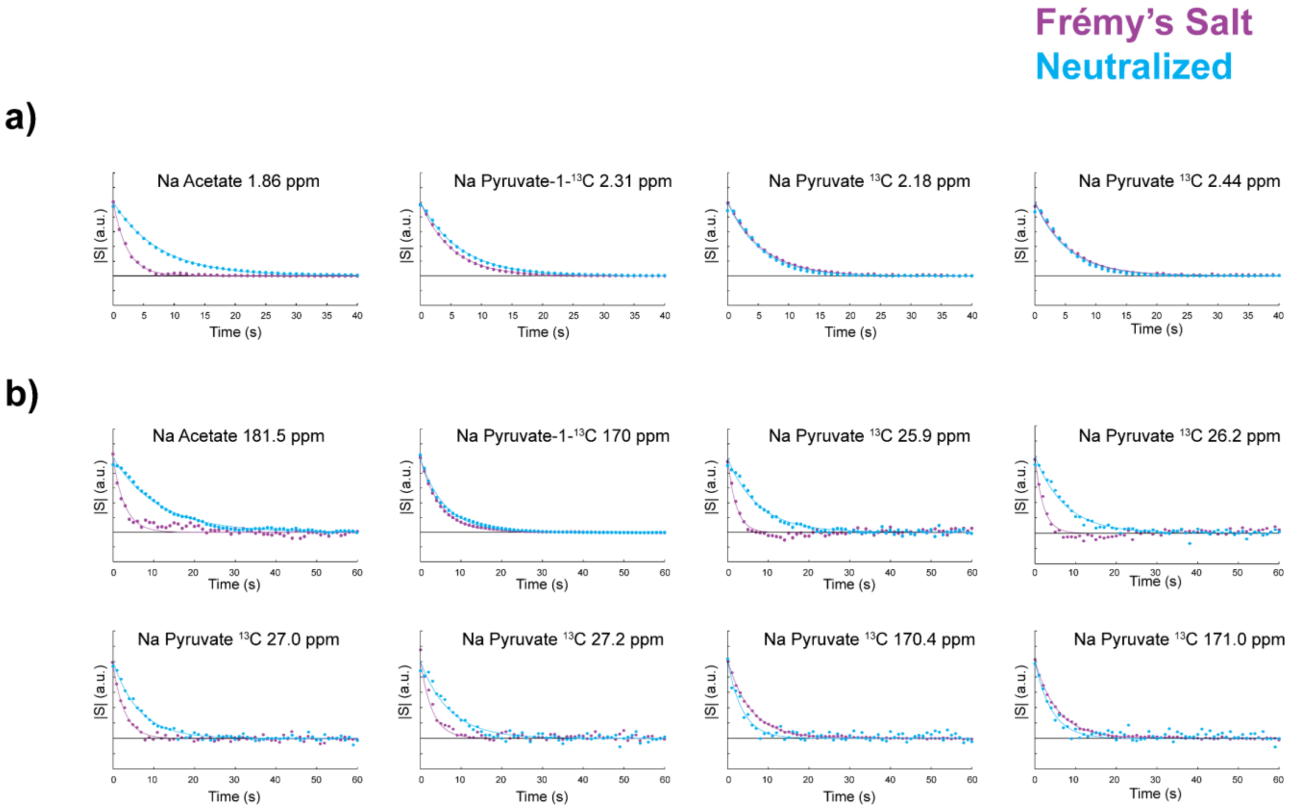

Figure S7: Absolute DDNP polarization decays with (grey) and without (purple) Frémy's salt neutralization, exponential fitting represented as solid lines. **a)** protons. **b)** carbons.

Schematic of the HySSS

The HySSS prototype's schematic view is shown in Fig. S8. It was utilized during the dDNP experiments to collect the hyperpolarized liquid mix it with the ascorbate solution and then transfer the mixture to the NMR tube. The entire fluid path is flanked by two magnetic plates (**14** in Fig. S8, further details in Fig. S9), which provide a magnetic field of 10 mT. The fluid path from the prototype to the NMR tube inside the spectrometer consists of a 3.2 mm OD 1 mm ID PTFE tube covered by a solenoid (2 turns per mm). Providing a magnetic field of > 37 mT (powered by a 24V DC power source capable to deliver a maximum of 20 A) and ending 15 cm before reaching the NMR tube inside the spectrometer. The solenoid was turned on only during liquid transfer to avoid heating of the tubing and the sample.

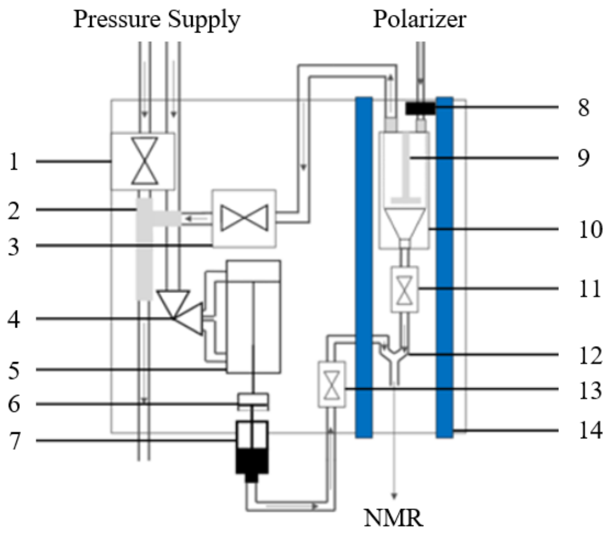

Figure S8: Schematic representation of the HySSS equipped with the collector/mixing chamber.

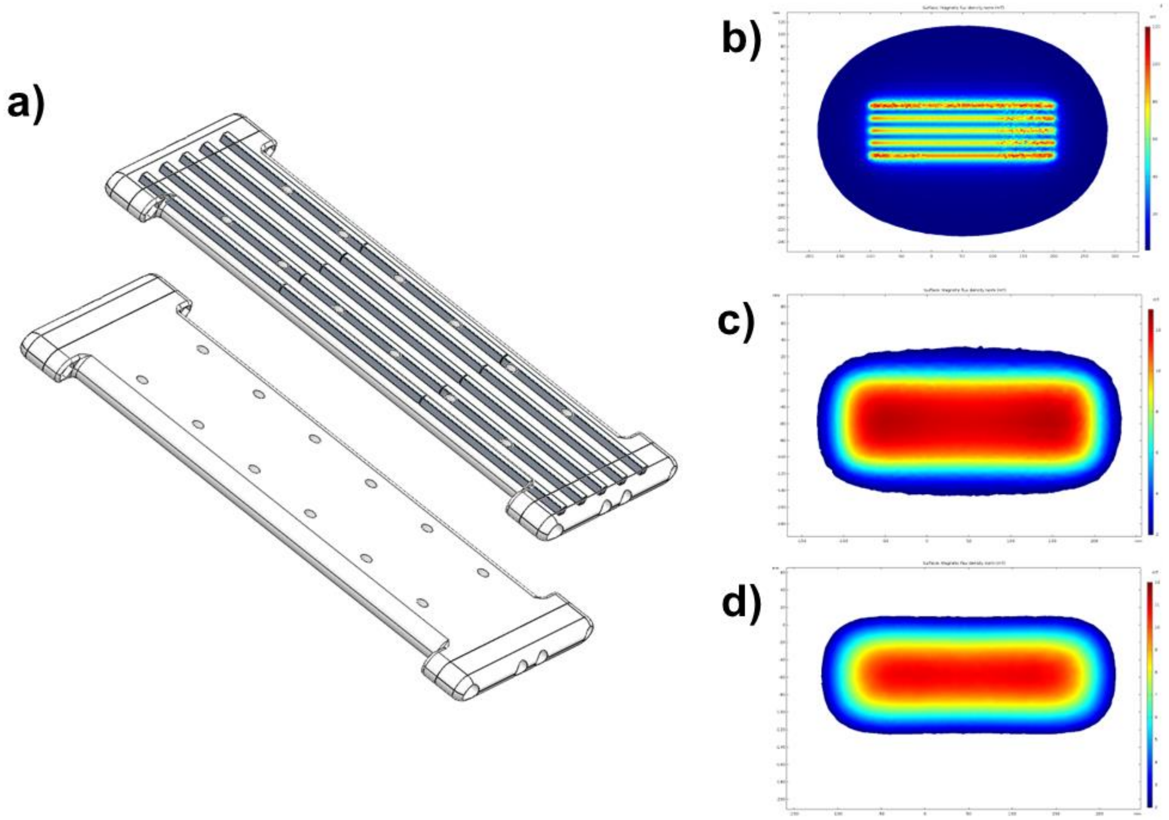

Figure S9: a) Representation of the magnetic plates with an x-ray scheme of the magnets inside. b-d) Magnetic field simulation at different distances from the plates: **b)** 0 mm, **c)** 25 mm and **d)** 50 mm.

Table S3: Detailed list of all the valves and further devices which are crucial in building the prototype

Nr.	Manufacturer parts number	Type
1	EVT317-5DO-02F-Q	3 port direct operated poppet valve
2	ZH10DLA-06-06-08	vacuum ejector
3	EVT317-5DO-02F-Q	3 port direct operated poppet valve
4	VUVG-LK10-M52-AT-M5-1H2L-S	5/2 port monostable solenoid valve
5	CD85N10-80-B	double acting single rod ISO cylinder
6		**custom made** connector (ISO cylinder to syringe)
7	RS PRO 181-1525	5 mL syringe
8	OPB350	optical flow through liquid sensor
9		**custom made** Vortex breaker
10		**custom made** Phase separator
11	VDW12HA	direct operated 2 port solenoid valve
12	QSMY-3	push-in Y-connector
13	VDW12HA	direct operated 2 port solenoid valve
14		magnetic plates

The sample collection and injection into the NMR tube consists of several steps:

1. A chase gas-driven sample entering the device is detected through a change in the current of the optical sensor **8** (OPB 350). The readout of the sensor values is performed via a microcontroller unit (Arduino Uno) operating according to a self-written program (*vide infra*).
2. Upon detection, the liquid enters the collector **10,** where degassing of the liquid proceeds through valves **1**, **3**, and a vacuum ejector **2**. At the same time, the ascorbate solution waiting in the collector is mixed with the arriving hyperpolarized solution.
3. After a degassing and mixing time of 2 s, 500 μL of the hyperpolarized and radical-free liquid is allowed to pass exit valve **11**.
4. As soon as **11** is closed, hydraulically-driven sample injection is triggered utilizing a pneumatic driver **5** coupled to syringe **7,** filled with a chase liquid. This results in a fast and controlled injection of the desired sample amount into the spectrometer. The sample and chase liquid are separate by an air cushion to prevent mixing.

The valves and the liquid detection were controlled by a microcontroller unit that operated with a home-written program code. Upon detecting the dissolved liquid, degassing and sample shuttling were triggered autonomously. Sample detection was performed by utilizing an optical liquid flow through a sensor connected according to Figure S10. Passing liquid led to a sudden change in the incoming current. Upon surpassing a defined and previously elaborated threshold value, the processes described in **1- 4** triggered after the delays listed in the sample code below:

```
float fillSeperator      = 1350;
float emptySeperator     =  240;
float timedegassing      =  750;

const int liquidPin_1    =  A2;

void setup() {
 pinMode (liquidPin_1,   INPUT);
 Serial.begin(115200);
}
void loop() {
 Serial.print ("\t Sensor Value_1: ");
 Serial.print (liquid_detection_1);
 liquid_detection_1 = analogRead(liquidPin_1);
 if (liquid_detection_1>575){
               VacuumControl();
             delay(emptySeperator);
             OutletControl();
 }
}
```

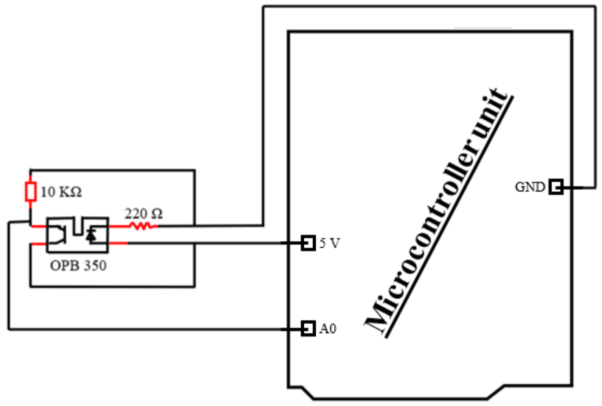

Figure S10: Illustration of the utilized optical flow through liquid detector in combination with the needed code for proper functionality. Through changing the resistor value of the potentiometer, the threshold value can be adjusted. According to the present setup the no liquid value was set to 300. Upon detection it would increase to 800.

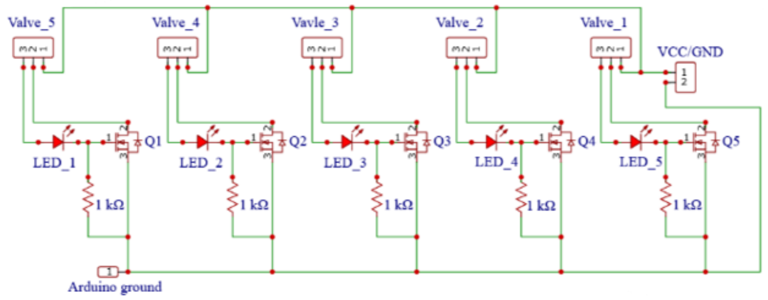

Figure S11: Schematic view of the utilized printed circuit board plate. Actuation of the valves was marked with embedded LED which gave optical feedback and made the process of valve actuation more visible.

As the microcontroller unit's output voltage was insufficient to supply the valves with their required power, a printed circuit board was designed for this particular purpose which, combined with a 24 V power source, made it possible to control the valves with the written microcontroller code. The following circuit diagram (Fig. S11) was designed with (www.easyeda.com) and constructed by (www.jlcpcb.com). Providing two independent circuits enabled the valve actuation through a combination of Transistors (RLZ34NPBF) and the microcontroller unit. The task of the microcontroller unit was to close the required circuits by applying the corresponding voltage to the specific gate.

The Arduino code for valve actuation is shown below. For the sake of clarity only necessary aspects of the Arduino code are displayed here.

```
float fillSeperator       = 1350;
float emptySeperator       =  240;
float timedegassing       =  750;
float retractSyringe       = 1000;

const int liquidPin_1      =  A0;
const int GateVacuumValve1 = 12;
const int GateVacuumValve2 = 11;
const int GateOutletValve  = 10;
const int GateSyringeValve = 9;
const int GatePneumatValve = 8;

int AV_NEO_trigger        = 4;        //trigger  for NMR to start signal acquisition

void setup() {
  pinMode (liquidPin_1,   INPUT);
```

```
   pinMode (GateVacuumValve1, OUTPUT);
   pinMode (GateVacuumValve2, OUTPUT);
   pinMode (GateOutletValve,  OUTPUT);
   pinMode (GateSyringeValve, OUTPUT);
   pinMode (GatePneumatValve, OUTPUT);
   pinMode (AV_NEO_trigger,   OUTPUT);

 Serial.begin(115200);
}

void loop() {
 Serial.print ("\t Sensor Value_1: ");
 Serial.print (liquid_detection_1);
 digitalWrite(AV_NEO_trigger,1);

 liquid_detection_1 = analogRead(liquidPin_1);
 if (liquid_detection_1 > 575) {
    delay(fillSeperator);
    digitalWrite (GateVacuumValve1,1);
    digitalWrite (GateVacuumValve2,1);
    delay(timedegassing);
    digitalWrite(AV_NEO_trigger,0);      //Start of  Signal acquisition
    digitalWrite (GateVacuumValve1,0);
    digitalWrite (GateVacuumValve2,0);
    digitalWrite(GateOutletValve,1);
    delay(emptySeperator);
    digitalWrite(GateOutletValve,0);
    digitalWrite(GateSyringeValve, 1);
    delay (50);
    digitalWrite(GatePneumatValve,1);
    delay(retractSyringe);
    digitalWrite(GatePneumatValve,0);
    ShutDown();
 }
}
```

References

1. Kress, T.; Che, K.; Epasto, L. M.; Kozak, F.; Negroni, M.; Olsen, G. L.; Selimovic, A.; Kurzbach, D., A novel sample handling system for dissolution dynamic nuclear polarization experiments. *Magnetic Resonance* **2021,** *2*, 387-394.
2. Negroni, M.; Guarin, D.; Che, K.; Epasto, L. M.; Turhan, E.; Selimovic, A.; Kozak, F.; Cousin, S.; Abergel, D.; Bodenhausen, G.; Kurzbach, D., Inversion of Hyperpolarized (13)C NMR Signals through Cross-Correlated Cross-Relaxation in Dissolution DNP Experiments. *J Phys Chem B* **2022,** *126* (24), 4599-4610.
3. Lumata, L.; Kovacs, Z.; Sherry, A. D.; Malloy, C.; Hill, S.; Van Tol, J.; Yu, L.; Song, L.; Merritt, M. E., Electron spin resonance studies of trityl OX063 at a concentration optimal for DNP. *Physical Chemistry Chemical Physics* **2013,** *15* (24), 9800-9807.
4. Heller, J.; Elgabarty, H.; Zhuang, B.; Sebastiani, D.; Hinderberger, D., Solvation of small disulfonate anions in water/methanol mixtures characterized by high-field pulse electron nuclear double resonance and molecular dynamics simulations. *The Journal of Physical Chemistry B* **2010,** *114* (22), 7429-7438.
5. Stoll, S.; Schweiger, A., EasySpin, a comprehensive software package for spectral simulation and analysis in EPR. *Journal of magnetic resonance* **2006,** *178* (1), 42-55.
